# Streptozotocin-Induced Diabetes in a Mouse Model (BALB/c) Is Not an Effective Model for Research on Transplantation Procedures in the Treatment of Type 1 Diabetes

**DOI:** 10.3390/biomedicines9121790

**Published:** 2021-11-29

**Authors:** Michal Wszola, Marta Klak, Anna Kosowska, Grzegorz Tymicki, Andrzej Berman, Anna Adamiok-Ostrowska, Joanna Olkowska-Truchanowicz, Izabela Uhrynowska-Tyszkiewicz, Artur Kaminski

**Affiliations:** 1 Foundation of Research and Science Development, 01-793 Warsaw, Poland; g.tymicki@wp.pl (G.T.); andrzejberman@gmail.com (A.B.); 2 Chair and Department of Histology and Embryology, Medical University of Warsaw, 02-004 Warsaw, Poland; akosowska@wum.edu.pl; 3 Department of Biochemistry and Molecular Biology, Centre of Postgraduate Medical Education, 01-813 Warsaw, Poland; anna.adamiok@cmkp.edu.pl; 4 Department of Transplantology and Central Tissue Bank, Medical University of Warsaw, 02-004 Warsaw, Poland; joanna.olkowska@gmail.com (J.O.-T.); izabela.uhrynowska-tyszkiewicz@wum.edu.pl (I.U.-T.); artur.kaminski@wum.edu.pl (A.K.)

**Keywords:** diabetic, T1D, mouse, streptozotocin, glucose, C-peptide, animal model, mouse

## Abstract

Type 1 diabetes (T1D) is characterized by the destruction of over 90% of the β-cells. C-peptide is a parameter for evaluating T1D. Streptozotocin (STZ) is a standard method of inducing diabetes in animals. Eight protocols describe the administration of STZ in mice; C-peptide levels are not taken into account. The aim of the study is to determine whether the STZ protocol for the induction of beta-cell mass destruction allows for the development of a stable in vivo mouse model for research into new transplant procedures in the treatment of type 1 diabetes. **Materials and methods:** Forty BALB/c mice were used. The animals were divided into nine groups according to the STZ dose and a control group. The STZ doses were between 140 and 400 mg/kg of body weight. C-peptide was taken before and 2, 7, 9, 12, 14, and 21 days after STZ. Immunohistochemistry was performed. The area of the islet and insulin-/glucagon-expressing tissues was calculated. **Results:** Mice who received 140, 160, 2 × 100, 200, and 250 mg of STZ did not show changes in mean fasting C-peptide in comparison to the control group and to day 0. All animals with doses of 300 and 400 mg of STZ died during the experiment. The area of the islets did not show any differences between the control and STZ-treated mice in groups below 300 mg. The reduction of insulin-positive areas in STZ mice did not exceed 50%. **Conclusions:** Streptozotocin is not an appropriate method of inducing a diabetes model for further research on transplantation treatments of type 1 diabetes, having caused the destruction of more than 90% of the β-cell mass in BALB/c mice.

## 1. Introduction

**Figure 1:**
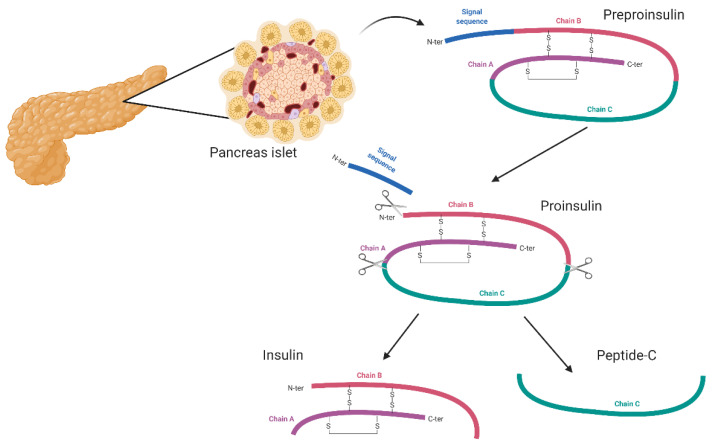
Production of insulin and C-peptide by the pancreatic islets. The diagram shows the processing of pre-proinsulin in the pancreatic islets resulting in the production of insulin and C-peptide. First, the signal sequence (blue) is cut from the primary pre-proinsulin chain, and the molecule is then cut into C-peptide (green) and insulin (pink-purple).

Diabetes mellitus is a common disease. It is characterized by a lack of insulin which results in hyperglycemia. Such a situation may lead to many complications such as neuropathy, nephropathy, and retinopathy as well as an increased risk of cardiovascular disease [1,2]. It is estimated that 463 million people are affected worldwide, and this number is still growing [3]. Diabetes mellitus has already become a severe global health-care problem [4]. It is predicted that by the year 2045, there will be around 629 million people aged between 20 and 79 years suffering from diabetes [5]. There are two types of diabetes: type 1 (T1D) and type 2 (T2D). T1D accounts for 10% of all people diagnosed with diabetes and is caused by autoimmune destruction of pancreatic insulin-producing beta-cells. This type of diabetes is characterized by the irreversible destruction of over 90% of the beta-cells that produce insulin (Figure 1). The main parameter for evaluating the severity of T1D is the study of metabolic markers—especially C-peptide. C-peptide is created during the production of insulin by the beta-cells of the pancreatic islets. Determination of C-peptide concentration allows for the assessment of the actual production of insulin by the pancreatic islets. In type 1 diabetes, pancreatic islets are destroyed, and insulin stores are significantly reduced, which translates into a low/undetectable concentration of C-peptide in the serum. However, in the case of type 2 diabetes, the tissues show initial insulin resistance—the level of C-peptide is high. C-peptide is a basic marker of T1D diagnostics because it shows a significantly higher stability in blood serum than insulin. The biological half-life of C-peptide is approximately 30 min in healthy subjects and is longer in diabetic patients [6]. This is a significantly longer half-life than that of insulin, which is about 3–5 min [6,7]. It is related to the metabolism of both proteins. Insulin secreted by the pancreas is metabolized in the liver by first-pass metabolism, while C-peptide has a negligible clearance rate in the hepatic organ. C-peptide is cleared in the peripheral circulation at a constant rate and insulin is removed alternately, making the direct measurement less consistent [7,8,9,10,11].

Due to such a rapid increase in new cases and the incomplete knowledge of the mechanisms causing type 1 diabetes, the medical community faces challenges in finding an effective method for diabetes treatment. A method that would allow patients to return to normal life without the need to constantly monitor glucose levels and administer insulin is urgently needed. Researchers of T1D predominantly perform their experiments on animal models with autoimmune diabetes and chemical ablation of pancreatic beta-cells [1]. Chemical induction of diabetes is the most common experimental model. Here, two pharmacological agents can be used: streptozotocin (STZ) and alloxan [12,13]. However, alloxan is not widely used due to its narrow diabetogenic dose—even a small overdose can cause general toxicity, especially to the kidneys [12]. Therefore, STZ is the most common method of chemically inducing diabetes in animal models today [1]. Streptozotocin is a broad-spectrum antibiotic derived from the *Streptomyces achromogenes* bacteria. It is a DNA alkylating agent and is often used as both an antimicrobial and an anticancer drug. However, due to its strong genotoxic effects, it is not recommended for common use. STZ is specifically cytotoxic to pancreatic β-cells and therefore, it is a substance primarily used as an agent for the induction of T1D in experimental models, especially in rodents [14,15,16]. STZ is a glucose analog that is selectively accumulated in pancreatic β-cells through the glucose transporter GLUT2 of the cell membrane [14,17]. The toxicity of STZ in beta-cells depends on the expression of GLUT2. After entering the beta-cells via the GLUT2 transporter, it causes DNA damage due to the DNA alkylating activity of its methyl nitrosourea moiety [14,18,19], which causes DNA fragmentation [14,20]. Next, the fragmented DNA activates poly (ADP-ribose) synthetase to repair the DNA. Poly ADP-ribosylation leads to the depletion of cellular NAD + and ATP [14,20,21]. This is manifested by dephosphorylation, which provides more substrates for xanthine oxidase and results in the formation of hydrogen peroxide and hydroxyl radicals [22,23], causing oxidative stress. Additionally, the presence of the N-methyl-N-nitrosourea side chain is capable of releasing nitric oxide, leading to mitochondrial dysfunction. The exact mechanism of cytotoxicity is still unclear. However, both apoptotic and necrotic β-cell death has been demonstrated [14,24,25]. In addition, changes caused by STZ cause mitochondrial dysfunction. STZ is diabetogenic due to its targeted glucose transporter protein (GLUT2), which is responsible for glucose movement across the membranes of pancreatic β-cells. The exact mechanism of cytotoxicity is still not fully understood. However, both apoptotic and necrotic β-cell death have been reported [14,26]. GLUT2 is mainly expressed in pancreatic β-cells and in the epithelial cells of kidney, intestine, and adult liver. Therefore, if sufficient doses are applied, STZ administration may lead to hepato- and nephrotoxicity along with its potential to damage β-cells. In pancreatic beta-cells, GLUT2 is involved in glucose-stimulated insulin secretion [27,28].

**Figure 2:**
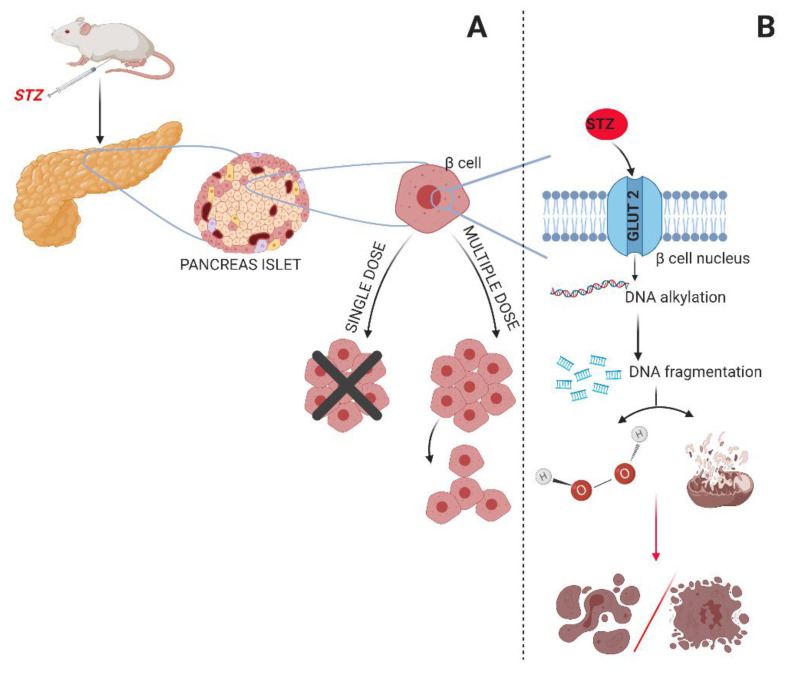
Diabetes induction model with STZ. (**A**) Mechanism of damage to pancreatic beta-cells with single or multiple doses of STZ. (**B**) Mechanism of action of STZ in the nucleus of β cells.

The mechanism of diabetes induction by STZ may vary depending on the administered dose. Thus, there are two frequently used methods for T1D induction. STZ is either administrated in a single, high-dose injection or several small doses. In the high-dose procedure, a single dose in the range of 100 to 200 mg/kg is used (Figure 2A). According to previous research, such a protocol produces massive pancreatitis resulting in the destruction of beta-cells with little or no insulin production [29,30,31,32,33,34,35]. Another method is characterized by the administration of multiple doses of 20 to 40 mg/kg/day. Similarly, such a procedure evokes pancreatitis [32,35,36,37,38]. During the development of islet inflammation, infiltration of macrophages in the pancreatic islet promotes the development of the destruction of the beta-cell mass dependent on the production of cytokines. Regardless of the method used, the parameters confirming the occurrence of complete destruction of the beta-cell mass should be monitored, e.g., the level of glucose and the value of c-peptide [1,26,31,32]. The currently available literature indicates that type T1D research models in mice can be diagnosed if the glucose level is increased above 300 mg/dL and the concentration of C-peptide is not higher than 0.6 ng/mL [13,39].

Eight protocols describe the preparation and administration of STZ mice in scientific publications (PubMed) and in scientific forums where scientists exchange knowledge and practical experience in conducting research (e.g., ResearchGate) [29,30,31,32,33,34,35,36,37,38,39,40]. A total of 106 studies were found via a PubMed search (word combination: mice, STZ, T1D). However, after adding the next word, “C-peptide”, to the search engine, there were four results [39,40,41,42]. As a result, the model of the pharmacological induction of diabetes appears to be unstable and far from perfect. A critical aspect of the articles published up to date is that they focus mainly on the administration of glycemic measurements, whereas the concentration of C-peptide, which is a pathognomonic factor enabling the definition of type 1 diabetes, is not taken into account. A lack of such elemental results in experiments, such as C-peptide testing, is a limiting factor for use in clinical applications. This study aims to determine whether the STZ protocol for the induction of beta-cell mass destruction allows for the development of a stable in vivo mouse model for research into new transplant procedures in the treatment of type 1 diabetes.

## 2. Materials and Methods

### 2.1. Materials

**Figure 3:**
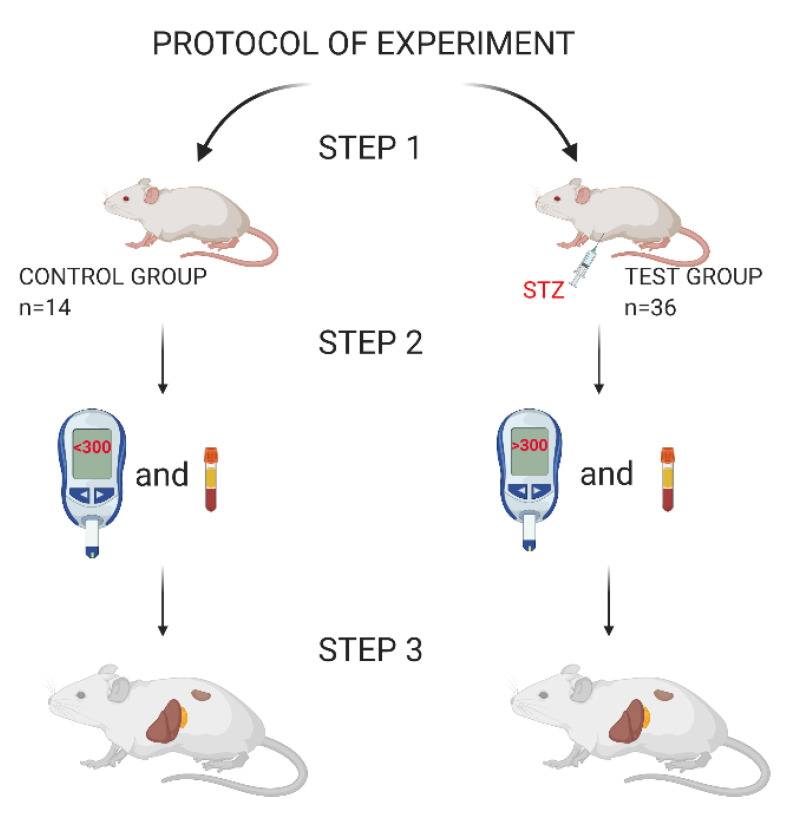
Experimental protocol. The entire experiment was divided into three stages of research. Step 1—assessment of the general health of the animals (bodyweight measurement/C-peptide blood sampling/blood glucose measurement at the experimental checkpoint); division of animals into appropriate groups; STZ injection in mice from the test groups. Step 2—measurement of glycemia and assessment of C-peptide concentration during the 21 days of the study. Step 3—taking the organs for histological analysis.

A total of 40 BALB/c mice were used for the experiment (Figure 3). All animals were male to eliminate the effects of hormone variation. The animals were 8 weeks old at the beginning of the study. They were fed ad libitum with fodder intended for laboratory mice (SNIFF M-Z distributed by Vivarii). The animals were kept under conditions of a higher standard of hygiene. This means that the lock system was insulated and the room was supplied with air through a ventilation system with a HEPA filter system. The animals were housed in individually ventilated cages. The feed, water, bedding, and animal houses were all sterilized. Environmental conditions were stable: temperature 20–22 °C, humidity 50–60%.

The experimental animals were divided into nine smaller groups according to the total dose of STZ administered and the dosing schedule (number of injections) to induce T1D and a control group. Each group consisted of 4 individuals. A total of six different concentrations of STZ were tested in the experiment. The highest dose was 400 mg/kg, and the lowest was 140 mg/kg of body weight. The dosing schedule is detailed in Table 1. All animal procedures were performed following the relevant guidelines and regulations. The research was conducted by the ARRIVE guidelines and approved by the Ethics Committee No. 1 of the Faculty of Biology, University of Warsaw, consent No. 461/2017.

As mentioned, to eliminate the defective batch of the drug, the dose of 200 mg/kg was repeated on two independent, active substances (group V1-Sigma-Aldrich; St. Louis, MO, USA cat.no: S0130-1G and group V2-Toku-E; USA; cat.no: S009-1g). This was the first stage of research. Both groups showed similar results, and thus the remaining study groups used only one drug (Toku-E; S009-1g). The induction of T1D by STZ consisted of the intraperitoneal injection of an appropriate dose of the drug dissolved in sodium citrate buffer (50 mM). STZ for injection is stable in this buffer. The solution was prepared fresh before administration, and its pH was 4.5. The drug was injected within 15 min of its preparation. For this purpose, a needle of 0.5 × 25 mm was used. The solution was kept at 4 °C throughout. The mice were fasted before injection. Only mice with a glucose concentration greater than 300 mg/dL and a C-peptide value less than 0.6 ng/mL were considered as sufficient models for further T1D study.

### 2.2. Elisa Kit

To determine the concentration of C-peptide in mice serum, measurements were taken before and 2, 7, 9, 12, 14, and 21 days after STZ administration. The determinations were performed using the ELISA test by ALPCO (catalog number: 80-CPTMS-E01; Salem, MA, USA). The amount of serum collected from the animals was sufficient and did not require dilution. The manufacturer specified that the sensitivity of the kit was 7.6 pM (0.03 ng/mL). However, careful analysis of the raw data (absorbance measurement) established that the results below 0.6 ng mL^−1^ can be considered breakpoints for diagnosing the destruction of more than 90% of the beta-cell mass in STZ-treated mice.

### 2.3. Glucose Measurement

Glucose measurement was performed with a glucometer using blood from a tail prick. A Wellion CALLA Light (Symphar Ltd.; Warsaw, Poland) glucometer was used to measure glucose concentration.

### 2.4. Immunohistochemistry

An immunohistochemistry investigation was performed on deparaffinized and dehydrated with xylene and graded ethanol sections. For histological evaluations, sections were stained with hematoxylin and eosin for 1 min.

For immunohistochemical analysis, pancreatic sections were dewaxed with xylene and dehydrated with ethanol. After antigen retrieval using 10 mM citric acid buffer (pH 6.0), the sections were blocked in 1% acetic acid for 10 min at room temperature and incubated for 1 h with insulin and glucagon antibodies diluted at 1:300. Alkaline phosphatase staining was performed with vector blue reagent (ImmPRESS-AP Horse Anti-Mouse IgG Polymer kit and Vector Blue AP substate, Vector Laboratories; Burlingame, CA, USA). The area and quantity of the glomerulus, islet, and insulin- and glucagon-expressing tissues area were calculated using ImageJ software (National Institutes of Health, Bethesda, MD, USA). In detail, the number of islets and glomerulus on the section was calculated using a multi-point tool; the area of islets and glomerulus was measured after their selection by a freehand selection tool; and insulin- and glucagon-positive regions were measured as above after applying a color threshold. The glucagon- and insulin-positive areas of STZ treated mice were related to the average islet area and the average islet area of the control mice.

### 2.5. Statistical Analysis

**Figure 4:**
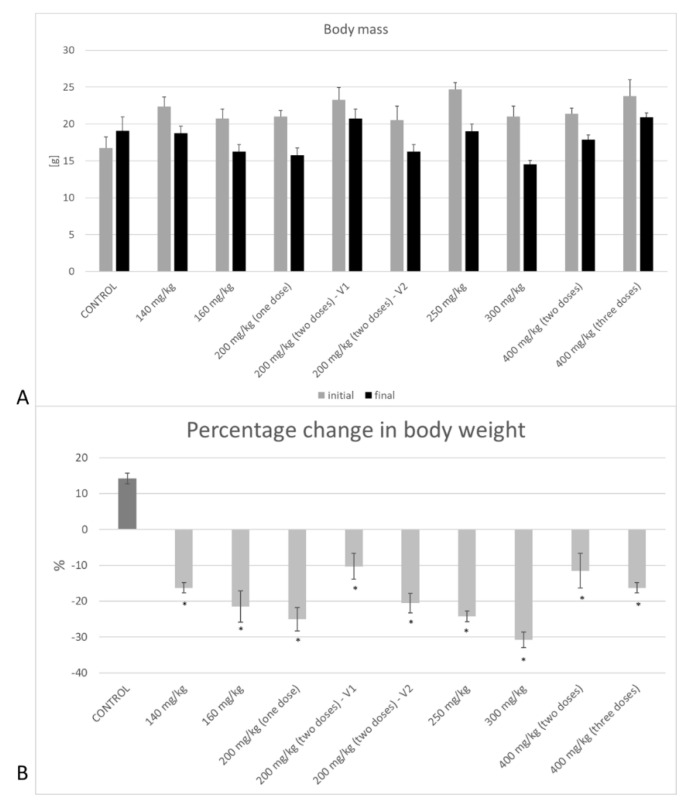
Change in body weight of the animals during the experiment. (**A**) The average weight of mice at time T0 on the day of starting the experiment and on the 21st day of the experiment (or on the day of death if the animals did not live to the end of the experiment). (**B**) Percentage change in body weight of tested groups with different STZ dose administration. Only the control group showed weight gain. On the other hand, all animals treated with STZ showed significant weight loss. Statistically significant values are marked with (*); *p* < 0.05.

**Figure 5:**
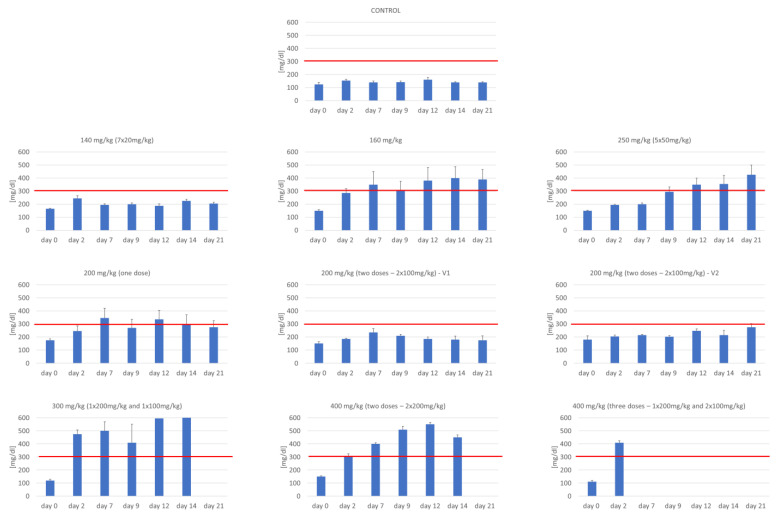
The glucose level in mice after intraperitoneal injection of STZ.

**Figure 6:**
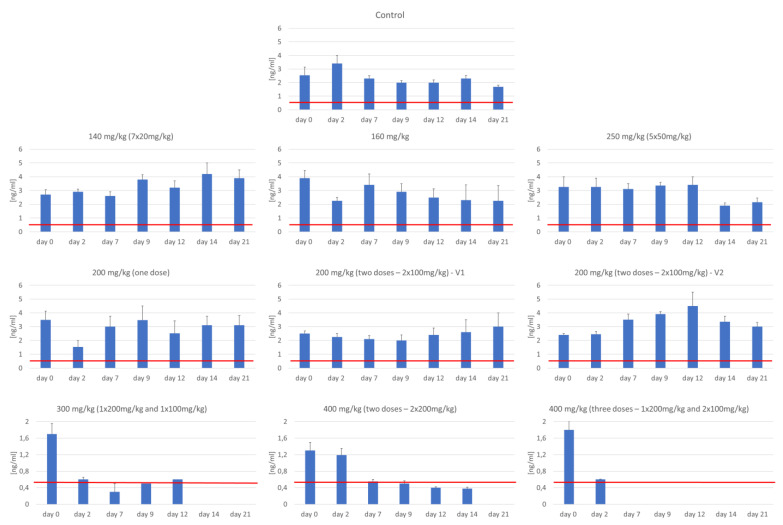
Level of C-peptide concentration.

The significant difference from the respective controls for each experimental test condition was assessed by a one-way analysis of variance (ANOVA) and the Dunnett test. The difference is significant if the *p*-value is less than 0.05. Statistical analysis of the data regarding Figures 4–6 was performed using GraphPad Prism V5.01 software (GraphPad Software Inc., La Jolla, CA, USA).

## 3. Results

### 3.1. Body Weight Control

The control group showed a 14.21% increase in body weight on average compared to the initial body weight. However, all experimental groups recorded a statistically significant decrease in body weight compared to the control group (*p* < 0.001). The mean weight loss among the study groups was 4.3 g ± 1.3 g (Figure 4). Initial mass was considered as the mass of the animals before the injection of STZ, whereas the final weight referred to the measurement made on the day of death of the animal.

### 3.2. Assessment of Glucose Concentration

Glucose was measured daily in the morning after overnight fasting. Figure 5 (and Table S1 in the Supplementary Material) shows the results at selected time points. The animals in the control group showed normal glucose levels throughout the experiment (the mean glucose level was 143.1 mg/dL) [43]. However, diabetes was found only in the presence of glycemia above 300 mg dL^−1^. The glucose level was elevated concerning the accepted norms in all the studied groups. Nevertheless, fasting glucose levels above 300 mg dL^−1^ were found only in six study groups (160, 250, 200 (one dose), 300, and 400 mg/kg STZ). It is worth noting that at the dose of 300 and 400 mg/kg, the animals were not able to live to the end of the experiment. The analysis of the results also showed fluctuations in glucose concentrations in animals from individual experimental groups (it was particularly visible when lower amounts of STZ were administered). These results were the individual response of the animals to the administered dose of the drug.

However, to support this hypothesis, we analyzed serum levels of C-peptide, which is a major diagnostic marker of beta-cell mass destruction.

### 3.3. Assessment of Serum C-Peptide Level

The group of mice who received 140, 160, 2 × 100, 200, and 250 mg of STZ/ kg of body weight did not show changes in mean fasting C-peptide in comparison to the control group and to day 0 in each group at the beginning of the experiment (Figure 6). The analysis showed a significantly lower level of C-peptide only in the groups in which STZ was used at a dose of 300 mg/kg of body weight and 400 mg/kg of body weight. (Figure 6). All animals from those groups died before the completion of the experiment.

### 3.4. STZ-Induced Diabetic Mice Showed Impairments in Pancreatic Islets, Glomeruli, and the Liver

#### 3.4.1. Islets Analysis

**Figure 7:**
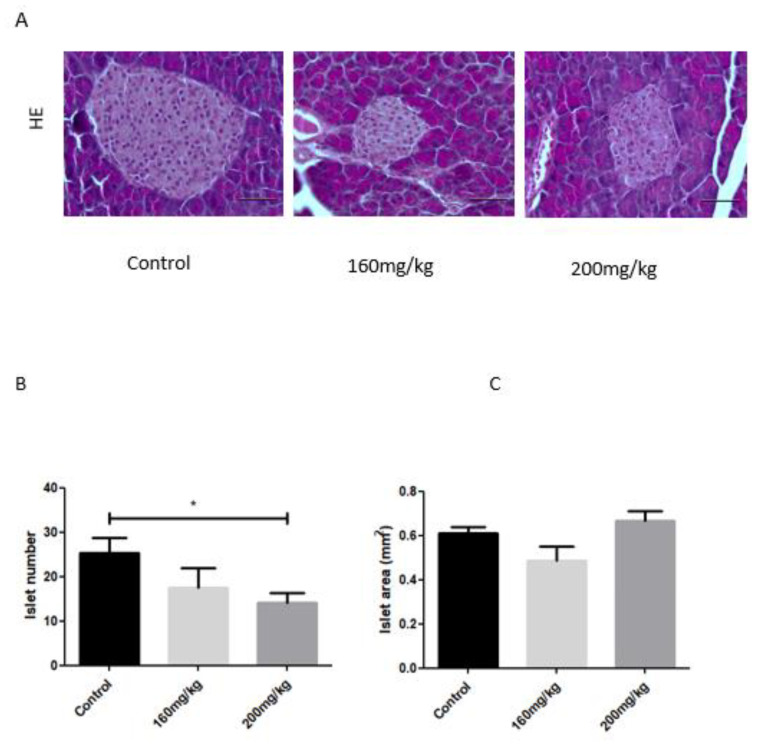
Pancreatic islet morphology in the pancreas of STZ-induced diabetic mice treated with STZ at different concentrations (140–250 mg kg^−1^). Representative hematoxylin and eosin staining of the islets of Langerhans was examined by light microscopy (magnification, ×10). (**A**). The quantifications of islet number and islet area were determined by ImageJ software (*n* = 3; *n* = 4; *n* = 6, shown in mm^2^) (**B**,**C**). Data are presented as mean SEM. STZ, streptozotocin; Control non-treated mice. Scale bar: 20 µm. Statistically significant values are marked with (*); *p* < 0.05.

The area of islets did not show any differences between control and STZ-treated mice in groups below 300 mg/kg of STZ (Figure 7C). The H&E staining showed that islet cells from control mice were smaller and more abundant than islets from STZ-treated mice (Figure 4A). The staining of H&E and insulin indicated the reduction of the pancreatic islets area in STZ-treated mice compared to that of control mice (Figure 7A,B). The reduction did not exceed 30% in islets number (Figure 7B).

**Figure 8:**
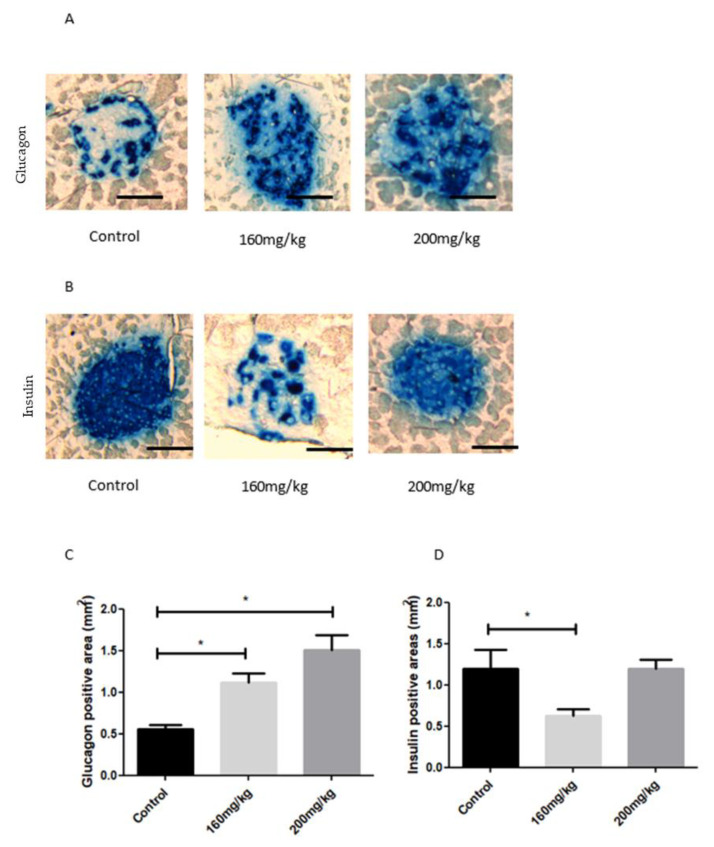
Insulin and glucagon production in the pancreas of STZ-induced diabetic mice treated with STZ at different concentrations (140–300 mg/kg). Insulin and glucagon staining of the islets of Langerhans examined by light microscopy are shown in blue (**A**,**B**). The quantifications of positive glucagon area and positive insulin area were determined by ImageJ software (*n* = 3; *n* = 4; *n* = 6, shown in mm^2^) (**C**,**D**). Data are presented as mean SEM, *p*-value < 0,05; Statistically significant values are marked with (*). STZ, streptozotocin; control non-treated mice. Scale bar: 20 µm.

We then evaluated the activity of α- and β-cells by staining the pancreatic sections with glucagon and insulin antibodies. The glucagon-positive area was more significant in STZ-treated mice and increased with the STZ dose (Figure 8A,C). The insulin-positive region of STZ mice was smaller than in the control group (Figure 8B,D). The reduction of insulin-positive areas between control and STZ mice did not exceed 50% (Figure 8D). Additionally, during the analysis of histological images, no inflammatory states were observed within the pancreatic islets.

#### 3.4.2. Kidney and Liver Analysis

A histological analysis assessing the pancreas, kidneys, and liver was also performed.

**Figure 9:**
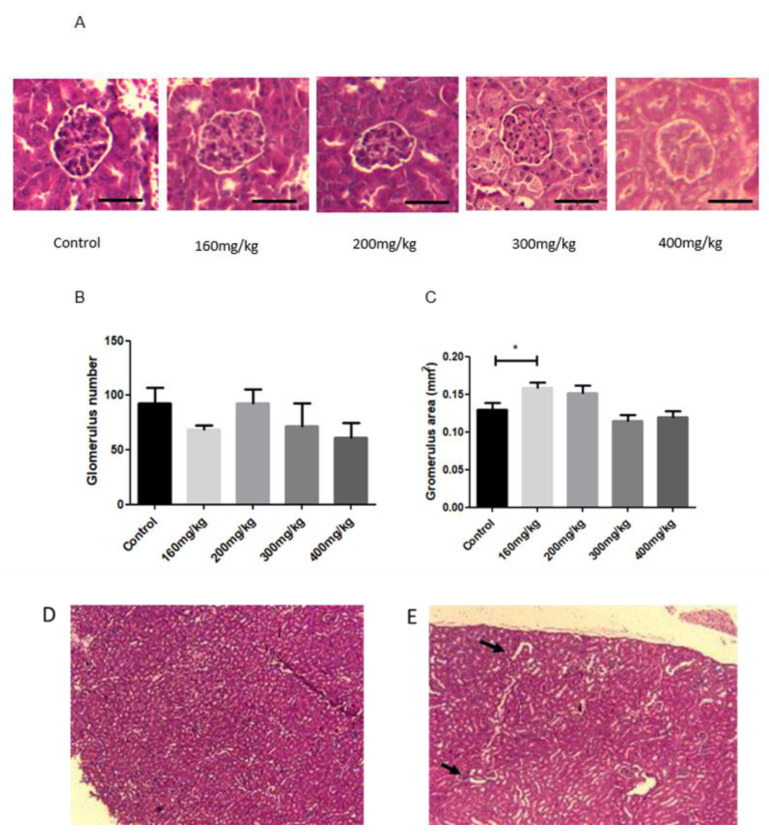
Glomerulus morphology in kidneys of STZ-induced diabetic mice treated with STZ at different concentrations (160–400 mg/kg). Representative hematoxylin and eosin staining of the glomerulus of kidneys was examined by light microscopy (magnification 10×) (**A**). The quantifications of glomerulus number and glomerulus area were determined by ImageJ software (*n* = 2; *n* = 3; *n* = 3; *n* = 4; *n* = 3, shown in mm^2^) (**B**,**C**). The tubular dilation appeared in STZ-treated mice (**E**, arrows) but not in control mice (**D**). Data are presented as mean SEM. STZ, streptozotocin; control non-treated mice. Statistically significant values are marked with (*); *p* < 0.05; Scale bar: 20 µm.

H&E staining of the kidney allowed us to analyze the glomerulus area and number. STZ-treated mice showed a reduction in the glomerulus compared to the control mice (Figure 9A,B). The area of the glomerulus was not changed in STZ-treated mice versus non-treated mice (Figure 9C). The tubular dilation was noted in STZ-treated mice (Figure 9D,E).

We also evaluated the histological analysis of the liver through H&E staining. The liver of STZ-induced diabetic mice showed dilution of the central vein and large necrotic areas.

## 4. Discussion

Many research teams believe that chemically induced diabetes provides a simple and relatively cheap model of diabetes in rodents, but it is a very demanding method. Mimicking type 1 diabetes should only be performed if over 90% of the beta-cell mass is destroyed. There is still no single, reliable protocol [29,30,31,32,33,34,35,36,37,38]. One of the most popular methods of inducing type 1 diabetes in small animals is the injection of streptozotocin.

All current methods of delivering STZ use intraperitoneal (IP) or intravenous (IV) injection [44]. Regardless of which injection method is used, the results are variable. The variability is related to the rate of STZ absorbance in the systemic circulation. Apart from the technical aspects of both techniques, neither of them has been shown to be advantageous so far. Generally, it may seem that the IV method is preferred in the literature; however, compared to IP, it is technically more difficult to perform. As mentioned, it does not provide a significant improvement over the obtained level of hyperglycemia [44,45,46,47,48]. Therefore, this study focused on one injection method, changing only the dosage in individual experimental groups.

The effectiveness of STZ-induced beta-cell mass destruction expressed as a high glucose concentration in serum is not in question, which was also shown in these studies. There are numerous research studies where blood glucose concentration was permanently elevated with the administration of STZ in rodent models [35,49,50,51] and on rhesus monkeys [52,53]. Nevertheless, an appropriate technique for the induction of over 90% beta-cell mass destruction by STZ administration is highly needed. There are no standardized (adjusted for specific animal models) protocols which can be considered reliable in inducing diabetes, which should be assessed as a C-peptide and glucose concentration. The team of Graham et al. [13] demonstrated in their analysis that the induction of diabetes in mice by STZ, apart from causing high sugars (based on these measurements they assessed the induction of diabetes), also caused severe and frequent side effects. Adverse reactions following STZ use included weight loss, respiratory failure, rapid and sudden changes in glycaemia leading to life-threatening hypoglycemia, and generalized poor condition of the animals. As the authors showed, the line between the induction of diabetes and the occurrence of complications was very thin [13]. Graham et al. demonstrated such difficulties when using pharmaceutical-grade STZ, for which chemical contamination and batch variability are limited and under constant control. The current one shows a significant variability depending on the given dose of STZ in terms of effectiveness and the severity of side effects [13]. These results also confirm the results of the present experiments. Therefore, it seems all the more important not to rely solely on glucose measurement in assessing the induction of damage to more than 90% of beta-cells by means of STZ.

Of course, when analyzing the available literature, we also encountered other markers indicating damage to the pancreatic islets. These include, but are not limited to, autoantibodies against β-cell antigens, including insulin, GAD, IA-2, and zinc transporter 8, and the GAD65 antibody [54]. Their presence in the serum indicates an ongoing immunological process. However, this does not necessarily mean that the beta-cells of the pancreatic islets are entirely damaged. There are reports in the literature that STZ can stimulate an autoimmune reaction by inducing the expression of MHC Class I or Class II antigens on the cell membrane. It is hypothesized that hyperglycemia in mice after multiple doses of STZ is due, at least in part, to the activation of an autoimmune response against pancreatic beta-cells [55,56] and lymphocytic infiltration within the pancreatic islets [57], which, however, was not observed in our histological results. It is worth noting that different strains of animals show different resistance to the effects of STZ, including genetic resistance. Some of them are resistant to the diabetic effects of low-dose streptozotocin therapy, while others are highly susceptible. However, even the sensitive ones require initial immunization prior to STZ infusion and sufficient time for the induction of autoimmune diabetes to be induced [55,57,58]. At this point, however, it is worth paying attention to the final goal of the research, which will be crucial for the relevance of detailed analyses. The induction of T1D with the occurrence of an immune reaction and the analysis of antibodies is essential for the research model to observe the course of T1D. On the other hand, in order to evaluate potential diabetes treatment methods, e.g., with stem cell therapy [59], transplantation of pancreatic islets [60], or a bioprinting model with beta-cells or pancreatic islets [61], the result of diabetes induction remains critical. Again, the key marker is the C-peptide concentration analysis. Therefore, considering the title of this manuscript, the following part of the work focuses on effective methods of inducing diabetes in a mouse model for metabolic and not for immunological purposes.

Following Leiter (2009), most studies upon murine models consider a glucose concentration over 300 mg/dL as a sign of T1D [62]. In our study, extreme values above 400 mg/dL were observed in a variant of the 300 mg/kg STZ dose administrated in two doses on the first and second day of the experimental trial. Such a concentration was noted on the second day of the experiment and remained above 300 mg/dL for 14 days. However, their general condition was so bad that the animals did not live to see the 14th day of the investigation. In the 300 mg/kg group, greater mortality occurred between the 7th and 9th day of the experiment. It appeared that a C-peptide concentration of 0.6 ng/mL, considered as a sign of diabetes, was found only in this variant of STZ dose administration. The concentration of C-peptide from day 2 to 14 oscillated around 0.5 ng/mL. An extremely high glucose concentration was also observed with the variant of the 400 mg/kg STZ dose injected in two doses, but in this case, the C-peptide concentration was above 0.6 ng/mL. The concentration of C-peptide indicating the complete destruction of the beta-cell mass appeared on the seventh day of the experimental trial, but after that time, most animals died. Unfortunately, there is a lack of studies on the murine T1D model, which combines the concentration of both glucose and C-peptide. Consequently, it is likely that in research where glucose concentration is the only indicator for T1D, some diagnoses might be biased. Nevertheless, the research group of Maldonado, who performed experiments on STZ-induced diabetic mice, evaluated C-peptide concentration. In this research, an increased C-peptide level was noted after transplantation of umbilical cord Wharton jelly cells (hUCWJCs), which resulted in insulin secretion by non-pancreatic cells [39]. Yet, diabetes dependent on the high destruction of the beta-cell mass of mice in this research was not confirmed by the evaluation of C-peptide concentration. It is noteworthy that the C-peptide effect on insulin depends on the stoichiometric ratio between them. Thus, a high concentration of C-peptide inhibits insulin, whereas a low concentration increases it [63,64]. In the latter case, C-peptide acts as an insulin mimetic. Moreover, it can modulate insulin signaling and takes part in multiple pathways [65]. Thus, it is highly recommended to verify the C-peptide concentration before the diagnosis of diabetes is made.

Streptozotocin is recognized as a nitrosourea compound which is also hepatotoxic, nephrotoxic, and often causes gastric ulceration [66]. Presented in this study, the effect of STZ on the morphology of kidneys is a relatively common phenomenon which results in the increased time duration and severity of hyperglycemia. There are several possible kidney lesions detected with STZ treatment, among which glomerulosclerosis, glomerular membrane thickening, arteriolar hyalinization, and widespread tubular necrosis frequently occur [67,68]. Considering mouse models, DBA/2(J) and KK/HIJ are highly susceptible to diabetic nephropathy with STZ treatment, whereas the C57BI/6J (B6) strain is more resistant to renal lesions. In this research, the BALB/c mice model expressed a tubular dilation, whereas such an outcome is not expressed in B6 mice [67]. Experiments performed on the rat model showed that animals with STZ-induced diabetes had an increased glomerular mesangial matrix by 65% [69]. Interestingly, substitutional doses of C-peptide prevented the matrix increase. This can be explained by the possible inhibitory effect of C-peptide on growth factors TGF-β1 and TNF-α [70]. In a clinical study of T1D patients, it was shown that those with a complete loss of insulin-producing cells and no C-peptide were more prone to the development of diabetic nephropathy [71]. The area of glomeruli in our mice model did not change after STZ treatment, which might be considered as indirect proof for the presence of C-peptide in mice sera.

Type 1 diabetes is also associated with numerous complications that can affect major organs. Thus, changes in body parameters are considered as important indicators of diabetogenic severity and catabolic effects [72]. In our study, we showed a significant body loss of STZ-induced T1D mice. This phenomenon was already found by Motyl et al. [72], where STZ-treated mice were found to have a greater magnitude of change in body mass and fat pad mass. The research group of Coe et al. worked on wild-type C57BL/6 mice which were injected with a low dose of STZ (40 µg/g body weight). Those animals expressed a 22% reduction of their mass in 5 weeks compared to the control of untreated mice [73]. Those results seem to be congruent with the observations made in our experiment, where in an experimental trial set to 3 weeks, mice lost from 10% to 30% of their mass depending on the applied dose of STZ. Nevertheless, mice used in our research were BALB/c, and the comparison of our data with those obtained by Coe et al. should not be made indiscriminately. Interestingly, the change of body mass on a different model of mice—Ins^2+/−^, which is programmed to be spontaneously diabetic at 5 weeks of age—is not that sharp. Predominantly, body mass reduction of STZ mice is recognized to be related to pathologies of bone and suppression in bone formation [74,75,76]. However, our data were not focused on the detection of possible sources of mass reduction.

Interesting results were also published by Koulmanda et al. (2003) [77], who found in their experimental studies that the use of streptozotocin can also be used in a wider spectrum of studies than just the induction of diabetes. Thus, STZ can be applied in preventing and reversing autoimmune diabetes in non-obese diabetic (NOD) mice. Those results indicated that although STZ prevents the development of destructive autoimmunity in NOD mice, its effect do not seem to be mediated simply through the death of β-cells. In the study of Hughes et al. (2002) [78], STZ caused impaired T-cell responses to islets’ antigens and mice were protected from diabetes development due to the apoptosis of islet cells. Similarly, Rayat et al. (2003) showed, on the same mice model, that the immunization of syngeneic islets while exposed to STZ prevents insulitis and the onset of autoimmune diabetes. Interestingly, a study by Yin et al. (2006) [79] where STZ with a high dose (160–170 mg/kg) was used to induce diabetes in C57BL/6 mice supports the possibility of the restoration of β-cell function in diabetic individuals. Thus, STZ-induced diabetes with the conditions presented by Yin et al. (2006) might be reversible. It must be kept in mind that STZ is an exogenous factor that is used to evoke the desired effect. However, as presented by Nicoletti et al. (2003) [80], other factors can not be underestimated during streptozotocin treatment. Those authors presented for the first time that IL-18 cytokine plays a crucial role in the development of murine diabetes when STZ is applied. It appeared that the treatment of the recombinant IL-18-binding protein: Fc (IL-18 bp: Fc) prevented diabetes and neither clinical nor histological signs of the disease appeared. Nevertheless, those results were observed while working on the C57BL/6 mice model, whereas BALB/c mice were employed in this study. Sun et al. (2005) [81], on the other hand, performed experiments with multiple low doses of STZ on BALB/c mice, and they found that oxidative macrophages are relevant in diabetes 1 development by their involvement in homeostasis between T-helper 1 (Th1) and T-helper 2 (Th2) activity.

The results presented in this study clearly show the effective induction of type 1 diabetes with a single dose of STZ, which practically resulted in the immediate death of animals due to systemic exhaustion. On the other hand, administration of lower doses caused an increase in glycemia that persisted for more than three days; however, C-peptide concentration was not low enough to diagnose the case as T1D. This may suggest that diabetes is induced, but it is not likely to mimic the T1D state. Herewith, we conclude that the STZ dose applied in this study did not entirely destroy the pancreatic β-cells, which is reflected by the high (0.6 ng/mL) C-peptide concentration. The particular problem is that injection of too low a dose of STZ does not completely damage the beta-cells of the pancreatic islets. At the same time, too high a dose causes the death of animals within a few days, which makes it impossible to conduct long-term research. It should be underlined that T1D induction with the STZ model is not an ideal solution but is still willingly used by researchers. As shown in this study, the BALB /c strain is not suitable for this type of experiment. Therefore, when planning such studies, the estimation of the C-peptide concentration should be considered to diagnose T1D models with the destruction of more than 90% of the mass of beta-cells in mice. As these studies have shown, high blood glucose alone may not directly reflect beta-cell mass destruction, which is desirable if any study wishes to show the restoration of beta-cell function by any treatment method. Further research should be also conducted on other types of mice, as there might be a higher type-specific response to STZ in different mice.

## 5. Conclusions

Based on the conducted research, we can conclude that streptozotocin is not an appropriate method of inducing a diabetes model, with the destruction of over 90% of the beta-cell mass in BALB/c mice for research into new transplant procedures for the treatment of type 1 diabetes.

## Figures and Tables

**Table 1:** Protocol of STZ administration to mice from a given experimental group.

No of Group	Group	Number (*n*)	Total Dose of STZ (mg/kg)	Dosage—Experiment Day (mg/kg b.w.)						
				1	2	3	4	5	6	7
1	400 mg/kg (three doses)	4	400	200	100	100	-	-	-	-
2	400 mg/kg (two doses)	4	400	200	-	-	-	-	-	200
3	300 mg/kg	4	300	200	100	-	-	-	-	-
4	250 mg/kg	4	250	50	50	50	50	50	-	-
5	200 mg/kg (one dose)	4	200	200	-	-	-	-	-	-
6	200 mg/kg (two doses)–V1	4	200	100	100	-	-	-	-	-
7	200 mg/kg (two doses)–V2	4	200	100	100	-	-	-	-	-
8	160 mg/kg	4	160	160	-	-	-	-	-	-
9	140 mg/kg	4	140	20	20	20	20	20	20	20

## Supplementary Materials

The following are available online at https://www.mdpi.com/article/10.3390/biomedicines9121790/s1, Table S1: Statistic analysis for the glucose level in mice after intraperitoneal injection of STZ.
