# Supplementary material for: Streptozotocin-Induced Diabetes in a Mouse Model (BALB/c) Is Not an Effective Model for Research on Transplantation Procedures in the Treatment of Type 1 Diabetes
Source: Biomedicines. 2021 Nov 29;9(12):1790. doi: 10.3390/biomedicines9121790 (PMC8698562; doi:10.3390/biomedicines9121790)
Supplement: Supplementary file 1 [file biomedicines-09-01790-s001.zip › biomedicines-1459596-supplementary.pdf]

biomedicines

Supplemental material

Streptozotocin-Induced Diabetes in Mouse Model (BALB/c) Is not an Effective Model for Research on Transplantation Procedures in Treatment of type 1 Diabetes

Michal Wszola [1,*,†], Marta Klak [1,*,†], Anna Kosowska [2], Grzegorz Tymicki [1], Andrzej Berman [1], Anna Adamiok-Ostrowska [3], Joanna Olkowska-Truchanowicz [4], Izabela Uhrynowska-Tyszkiewicz [4] and Artur Kaminski [4]

[1] Foundation of Research and Science Development, 01-793 Warsaw, Poland; g.tymicki@wp.pl (G.T.); andrzejberman@gmail.com (A.B.)
[2] Chair and Department of Histology and Embryology, Medical University of Warsaw, 02-004 Warsaw, Poland; akosowska@wum.edu.pl
[3] Department of Biochemistry and Molecular Biology, Centre of Postgraduate Medical Education, 01-813 Warsaw, Poland; anna.adamiok@cmkp.edu.pl
[4] Department of Transplantology and Central Tissue Bank, Medical University of Warsaw, 02-004 Warsaw, Poland; joanna.olkowska@gmail.com (J.O.-T.); izabela.uhrynowska-tyszkiewicz@wum.edu.pl (I.U.-T.); artur.kaminski@wum.edu.pl (A.K.)
* Correspondence: michal.wszola@fundacjabirn.pl (M.W.); marta.klak@fundacjabirn.pl (M.K.)
† Equal contribution.

Citation: Wszola, M.; Klak, M.; Kosowska, A.; Tymicki, G.; Berman, A.; Adamiok-Ostrowska, A.; Olkowska-Truchanowicz, J.; Uhrynowska-Tyszkiewicz, I.; Kaminski, A. Streptozotocin Induced Diabetes in a Mouse Model (BALB/c) Is Not an Effective Model for Research on Transplantation Procedures in the Treatment of Type 1 Diabetes. Biomedicines 2021, 9, 1790. https://doi.org/10.3390/biomedicines9121790

Academic David G. Alleva

Received: 27 October 2021
Accepted: 25 November 2021
Published: 29 November 2021

Publisher's Note: MDPI stays neutral with regard to jurisdictional claims in published maps and institutional affiliations.

Copyright: © 2021 by the authors. Submitted for possible open access publication under the terms and conditions of the Creative Commons Attribution (CC BY) license (https://creativecommons.org/licenses/by/4.0/).

Abstract: Type 1 diabetes(T1D) is characterized by the deststruction of over 90% of the β-cells. C-peptide is a parameter for evaluating T1D. Streptozotocin(STZ) is a standard method of inducing diabetes in animals. Eight protocols describing the administration of STZ in mice. C-peptide levels aren't taken into account. The aim of the study is to determine whether the STZ protocol for the induction of beta-cell mass destruction allows for the development of a stable in vivo mouse model for research into new transplant procedures in the treatment of type 1 diabetes. **Materials&methods:**40 BALB/c mice were used. The animals were divided into nine groups according to the STZ dose and a control group. The STZ doses were between 140-400 mg/kg body weight. C-peptide was taken before and 2,7,9,12,14,21 days after STZ. Immunohistochemistry was performed. The area of the islet and insulin-/glucagon-expressing tissues was calculated. **Results:** Mice who received 140,160,2x100,200,250 mg of STZ didn't show changes in mean fasting c-peptide in comparison to the control group and to day 0. All animals from 300,400mg of STZ died during the experiment. The area of islets didn't show any differences between control and STZ-treated mice in groups below 300mg. The reduction of insulin-positive areas in STZ mice didn't exceed 50%. **Conclusion:** Streptozotocin is not an appropriate method of inducing a diabetes model with the destruction of more than 90% of the β-cell mass in Balb/c mice for further research on transplantation treatment of type 1 diabetes.

Keywords: diabetic; T1D; mouse; streptozotocin; glucose; C-peptide; animal model; mouse

Table S1. Statistic analysis for the glucose level in mice after intraperitoneal injection of STZ.

CONTROL	Day 0	Day 2	Day 7	Day 9	Day 12	Day 14	Day 21
Day 0	-	NS	NS	NS	NS	NS	NS
Day 2	NS	-	NS	NS	NS	NS	NS
Day 7	NS	NS	-	NS	NS	NS	NS
Day 9	NS	NS	NS	-	NS	NS	NS
Day 12	NS	NS	NS	NS	-	NS	NS
Day 14	NS	NS	NS	NS	NS	-	NS
Day 21	NS	NS	NS	NS	NS	NS	-

140 mg/kg	Day 0	Day 2	Day 7	Day 9	Day 12	Day 14	Day 21
Day 0	-	0.0003	NS	0.0373	NS	0.0050	0.0210
Day 2	0.0003	-	0.0315	0.0467	0.0115	NS	NS
Day 7	NS	0.0315	-	NS	NS	NS	NS
Day 9	0.0373	0.0467	NS	-	NS	NS	NS
Day 12	NS	0.0115	NS	NS	-	NS	NS
Day 14	0.0050	NS	NS	NS	NS	-	NS
Day 21	0.0210	NS	NS	NS	NS	NS	-

160 mg/kg	Day 0	Day 2	Day 7	Day 9	Day 12	Day 14	Day 21
Day 0	-	NS	NS	NS	0.0398	0.0288	0.0354
Day 2	NS	-	NS	NS	NS	NS	NS
Day 7	NS	NS	-	NS	NS	NS	NS
Day 9	NS	NS	NS	-	NS	NS	NS
Day 12	0.0398	NS	NS	NS	-	NS	NS
Day 14	0.0288	NS	NS	NS	NS	-	NS
Day 21	0.0354	NS	NS	NS	NS	NS	-

250 mg/kg	Day 0	Day 2	Day 7	Day 9	Day 12	Day 14	Day 21
Day 0	-	NS	NS	0.0388	0.0077	0.0060	0.0004
Day 2	NS	-	NS	NS	0.0357	0.0285	0.0022
Day 7	NS	NS	-	NS	0.0462	0.0371	0.0030
Day 9	0.0388	NS	NS	-	NS	NS	NS
Day 12	0.0077	0.0357	0.0462	NS	-	NS	NS
Day 14	0.0060	0.0285	0.0371	NS	NS	-	NS
Day 21	0.0004	0.0022	0.0030	NS	NS	NS	-

200 mg/kg (one dose)	Day 0	Day 2	Day 7	Day 9	Day 12	Day 14	Day 21
Day 0	-	NS	NS	NS	NS	NS	NS
Day 2	NS	-	NS	NS	NS	NS	NS
Day 7	NS	NS	-	NS	NS	NS	NS
Day 9	NS	NS	NS	-	NS	NS	NS
Day 12	NS	NS	NS	NS	-	NS	NS
Day 14	NS	NS	NS	NS	NS	-	NS
Day 21	NS	NS	NS	NS	NS	NS	-

200 mg/kg (two dose) – V1	Day 0	Day 2	Day 7	Day 9	Day 12	Day 14	Day 21
Day 0	-	NS	0.0020	0.0267	NS	NS	NS
Day 2	NS	-	0.0326	NS	NS	NS	NS
Day 7	0.0020	0.0326	-	NS	0.0424	0.0398	0.0303
Day 9	0.0267	NS	NS	-	NS	NS	NS
Day 12	NS	NS	0.0424	NS	-	NS	NS
Day 14	NS	NS	0.0398	NS	NS	-	NS
Day 21	NS	NS	0.0303	NS	NS	NS	-

200 mg/kg (two dose) – V2	Day 0	Day 2	Day 7	Day 9	Day 12	Day 14	Day 21
Day 0	-	NS	NS	NS	0.0378	NS	0.0042
Day 2	NS	-	NS	NS	NS	NS	0.0295
Day 7	NS	NS	-	NS	NS	NS	NS
Day 9	NS	NS	NS	-	NS	NS	0.0199
Day 12	0.0378	NS	NS	NS	-	NS	NS
Day 14	NS	NS	NS	NS	NS	-	NS
Day 21	0.0042	0.0295	NS	0.0199	NS	NS	-

300 mg/kg	Day 0	Day 2	Day 7	Day 9	Day 12	Day 14	Day 21
Day 0	-	0.0021	0.0013	0.0160	0.0061	0.0050	-
Day 2	0.0021	-	NS	NS	NS	NS	-
Day 7	0.0013	NS	-	NS	NS	NS	-
Day 9	0.0160	NS	NS	-	NS	NS	-
Day 12	0.0061	NS	NS	NS	-	NS	-
Day 14	0.0050	NS	NS	NS	NS	-	-
Day 21	-	-	-	-	-	-	-

400 mg/kg (two doses)	Day 0	Day 2	Day 7	Day 9	Day 12	Day 14	Day 21
Day 0	-	0.0003	<0.0001	<0.0001	<0.0001	<0.0001	-
Day 2	0.0003	-	0.0046	<0.0001	<0.0001	0.0018	-
Day 7	<0.0001	0.0046	-	0.0051	0.0043	NS	-
Day 9	<0.0001	<0.0001	0.0051	-	NS	NS	-
Day 12	<0.0001	<0.0001	0.0043	NS	-	NS	-
Day 14	<0.0001	0.0018	NS	NS	NS	-	-
Day 21	-	-	-	-	-	-	-

400 mg/kg (three doses)	Day 0	Day 2	Day 7	Day 9	Day 12	Day 14	Day 21
Day 0	-	<0.0001	-	-	-	-	-
Day 2	<0.0001	-	-	-	-	-	-
Day 7	-	-	-	-	-	-	-
Day 9	-	-	-	-	-	-	-
Day 12	-	-	-	-	-	-	-
Day 14	-	-	-	-	-	-	-
Day 21	-	-	-	-	-	-	-
